# Disseminated *Mycobacterium avium* Complex Infection in a Patient Treated With Immunosuppressants

**DOI:** 10.14309/crj.0000000000001033

**Published:** 2023-04-19

**Authors:** Yuki Ito, Daisuke Watanabe, Sayaka Ikeda, Norihiro Okamoto, Haruka Miyazaki, Eri Tokunaga, Yuna Ku, Makoto Ooi, Namiko Hoshi, Yuzo Kodama

**Affiliations:** 1 Division of Gastroenterology, Department of Internal Medicine, Kobe University Graduate School of Medicine, Kobe, Japan

**Keywords:** mycobacterium avium complex (MAC), disseminated MAC, mycophenolate mofetil (MMF)

## Abstract

*Mycobacterium avium* complex (MAC) is an important cause of opportunistic infections in immunosuppressed hosts, such as patients with HIV infection and solid organ transplant recipients. MAC disease usually presents in 4 distinct clinical categories: chronic pulmonary disease, disseminated disease, skin/soft-tissue infection, and superficial lymphadenitis. However, clinical reports on gastrointestinal (GI) MAC disease are rare, especially in patients without HIV infection or a history of organ transplantation. We describe a case of non-HIV-associated GI MAC disease in a patient with long-term mycophenolate mofetil use. In this case, MAC organisms in the GI tract and ascites were observed. Endoscopy revealed a unique colonic image with large, deep epithelial denudations. This suggests that apart from patients with HIV infection or transplant recipients, those treated with immunosuppressants can have disseminated MAC. Therefore, internal physicians need to monitor patients undergoing mycophenolate mofetil immunosuppressant therapy.

## INTRODUCTION

Mycobacterial species other than *Mycobacterium tuberculosis* and *Mycobacterium leprae* are classified as nontuberculous mycobacteria (NTM). The *Mycobacterium avium* complex (MAC) includes the most common species associated with human infectious diseases. The MAC consists of a large number of *Mycobacterium* species, including *M. avium* and *Mycobacterium intracellulare*.^1^ MAC organisms that are prevalent in normal environments can be isolated from water, house dust, and soil.^1^ However, even if hosts are exposed to these bacteria, those with normal immunity can defend themselves against them. By contrast, MAC species are recognized as important causes of opportunistic infection in immunosuppressed hosts. Diseases associated with NTM, including MAC, mainly present in 4 distinct clinical categories in humans: (i) chronic pulmonary disease, (ii) disseminated disease, (iii) skin/soft-tissue infection, and (iv) superficial lymphadenitis.^1^ However, clinical reports of gastrointestinal (GI) MAC resulting from disseminated MAC are rare.

We describe a case of non-HIV-associated GI MAC disease in a patient on long-term mycophenolate mofetil (MMF; CellCept) therapy. During the clinical course, the patient was found to have MAC organisms in the GI tract and ascites, presenting a unique colonic image on endoscopic examination.

## CASE REPORT

**Figure 1.**
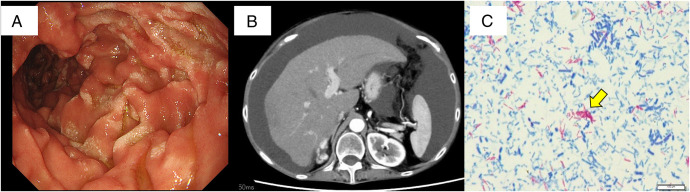
(A) Upper gastrointestinal endoscopic findings showing diffuse swollen white villi in the duodenum. (B) Significant ascites is detected by abdominal computed tomography. (C) Acid-fast staining of ascitic fluid showing many acid-fast bacilli (small purple elongated structures; yellow arrow).

**Figure 2.**
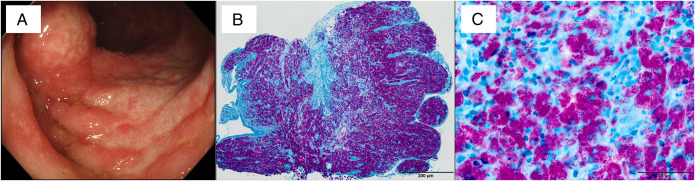
(A) Lower gastrointestinal endoscopic findings showing large deep epithelial denudations surrounded by an erythematous mucosa in the ascending colon. (B) A low-magnification (40×) image of Ziehl-Neelsen staining of the colon biopsy specimen showing massive infiltration by *Mycobacteria* phagocytosed by intestinal macrophages in the lamina propria mucosae. (C) A high-magnification (400×) image of Ziehl-Neelsen staining of the colon biopsy specimen showing many small purple elongated structures (*Mycobacterium* species) phagocytosed by intestinal macrophages.

A 66-year-old woman was referred to our department because of weight loss, watery diarrhea, hypoalbuminemia, and zinc deficiency. Laboratory data were remarkable, with a low lymphocyte count (265 cells/μL). Physical examination was normal, and her medical history was significant (she had epidermolysis bullosa acquisita that had been treated with MMF [2 g/d] and prednisolone in the past 2 years). We conducted upper GI endoscopy, which showed diffuse swelling of the white villi in the duodenum (Figure 1). Histopathological examination of a duodenal biopsy specimen revealed dilated lymphatic vessels in the villi. The patient was diagnosed with intestinal lymphangiectasia and was treated with an elemental diet. Approximately 1.5 years later, bilateral lower extremity edema and abdominal distension appeared. Laboratory examination revealed severe lymphocytopenia (200 cells/μL) and hypoalbuminemia (2.1 g/dL). Computed tomography revealed ascites that was not evident 1 year earlier (Figure 1). We then increased the prednisolone dose from 6 to 20 mg daily for lymphangiectasia and additionally controlled the ascites using a diuretic agent and abdominal paracentesis. Four months later, she visited our hospital with complaints of severe diarrhea and difficulty walking and was admitted. Physical examination on admission revealed low blood pressure (71/56 mm Hg) and severe abdominal distension without pain. Laboratory data revealed an extremely low CD4-positive cell count of only 35 cells/μL (lymphocyte count, 168 cells/μL), although her serum HIV antibody test was negative. Computed tomography scan revealed diffuse colonic wall thickening and increased ascites compared with the previous scan. Colonoscopy revealed large deep epithelial denudations surrounded by an erythematous mucosa (Figure 2). Surprisingly, biopsy specimens from the colonic mucosa stained by the Ziehl-Neelsen method revealed many acid-fast bacilli phagocytosed by macrophages (Figure 2). Acid-fast bacilli were detected in the ascitic fluid by acid-fast staining (Figure 1), and the presence of *M. avium* was confirmed by polymerase chain reaction and culture. Furthermore, a culture study confirmed the presence of *M. avium* in the blood, and a diagnosis of peritonitis and colitis because of disseminated MAC was made. Soon after the diagnosis was made, MMF was discontinued and oral ethambutol, rifabutin, and azithromycin were administered. However, her overall condition deteriorated, albeit with a slight improvement in the lymphocyte count, and she died on day 32 of hospitalization.

## DISCUSSION

MAC is the most common species causing pulmonary and disseminated NTM infection.^1,2^ In pulmonary diseases (approximately 90% of NTM infections involve the pulmonary system), inhalation of infectious aerosols is likely to be the primary mode of transmission.^1,2^ However, MAC is probably acquired through the GI tract in patients with HIV. Mechanistically, it begins when colonizing MAC in the GI tract adheres to the gut mucosa, penetrates the lamina propria, and is phagocytosed by the macrophages.^3^

Although no case report of disseminated MAC infection solely arising from corticosteroid intake has been identified, it is widely recognized that corticosteroids increase the risk of nontuberculous mycobacteria infections.^4^ Meanwhile, the incidence and prevalence of NTM infections have increased in solid organ transplant recipients but declined in those with HIV because of the widespread availability of antiretroviral therapy.^5^ However, mortality from disseminated MAC in patients with HIV remains high.^5^ This may be because there are clinical differences in the typical presentations of MAC infections between patients with HIV and solid organ transplant recipients. HIV-related MAC infections more commonly present with disseminated MAC,^6,7^ particularly when CD4-positive T-cell counts decrease to less than 50 cells/mm^3^.^8^ In this case, despite a negative serum test for HIV, the patient had extremely low counts of CD4-positive T cells, probably making the patient prone to MAC dissemination.

MMF is used to treat a wide range of autoimmune diseases including epidermolysis bullosa acquisita. MMF is a prodrug of mycophenolic acid, an inhibitor of inosine monophosphate dehydrogenase. Through this mechanism, MMF inhibits the formation of guanine nucleotides. Unlike many other cell types, lymphocytes rely solely on the de novo pathway to generate guanine nucleotides,^9^ and MMF preferentially inhibits the inosine monophosphate dehydrogenase isoform found in B and T lymphocytes.^10^ Therefore, it decreases lymphocyte proliferation and suppresses both antibody-mediated and cell-mediated responses.^10^ In addition, there is a particular concern that MMF lowers CD4 counts, which is particularly evident in transplant recipients.^11^ The patient in this case had extremely low counts of CD4-positive cell subsets compared with the total counts of lymphocytes, potentially triggering colitis and peritonitis because of MAC dissemination.

In conclusion, disseminated MAC disease can occur in patients treated with immunosuppressants, apart from patients with HIV or transplant recipients. However, if MAC disease is not suspected, the diagnosis is difficult. Therefore, when a patient undergoes immunosuppressive therapy (eg, MMF), CD4-positive T-cell counts should be monitored. In addition, when a patient has intestinal or septic symptoms, disseminated MAC disease should be considered in the differential diagnosis.

## DISCLOSURES

Author contributions: All authors collected the data and wrote the manuscript. D. Watanabe is the article guarantor.

Financial disclosure: None to report.

Informed consent was obtained for this case report.
